# Machine learning algorithm validation with a limited sample size

**DOI:** 10.1371/journal.pone.0224365

**Published:** 2019-11-07

**Authors:** Andrius Vabalas, Emma Gowen, Ellen Poliakoff, Alexander J. Casson

**Affiliations:** 1 Materials, Devices and Systems Division, School of Electrical and Electronic Engineering, The University of Manchester, Manchester, England, United Kingdom; 2 School of Biological Sciences, The University of Manchester, Manchester, England, United Kingdom; Instituto Nacional de Medicina Genomica, MEXICO

## Abstract

Advances in neuroimaging, genomic, motion tracking, eye-tracking and many other technology-based data collection methods have led to a torrent of high dimensional datasets, which commonly have a small number of samples because of the intrinsic high cost of data collection involving human participants. High dimensional data with a small number of samples is of critical importance for identifying biomarkers and conducting feasibility and pilot work, however it can lead to biased machine learning (ML) performance estimates. Our review of studies which have applied ML to predict autistic from non-autistic individuals showed that small sample size is associated with higher reported classification accuracy. Thus, we have investigated whether this bias could be caused by the use of validation methods which do not sufficiently control overfitting. Our simulations show that K-fold Cross-Validation (CV) produces strongly biased performance estimates with small sample sizes, and the bias is still evident with sample size of 1000. Nested CV and train/test split approaches produce robust and unbiased performance estimates regardless of sample size. We also show that feature selection if performed on pooled training and testing data is contributing to bias considerably more than parameter tuning. In addition, the contribution to bias by data dimensionality, hyper-parameter space and number of CV folds was explored, and validation methods were compared with discriminable data. The results suggest how to design robust testing methodologies when working with small datasets and how to interpret the results of other studies based on what validation method was used.

## Introduction

Generally, the larger the dataset the greater statistical power for pattern recognition [1]. Large datasets are also becoming more common, partly because the data is increasingly gathered by cheap and widely available methods such as Internet of things (IoT) devices. Databases such as the UK Biobank [2] are aggregating data from more than 500,000 people to enable very large-scale data analysis, provided that the desired analysis is supported by the data available in the database.

Unfortunately, such techniques and large databases are of less use for traditional hypothesis driven research. Advances in neuroimaging, genomic, motion-tracking, eye-tracking and many other technology-based data collection methods have led to many datasets, which frequently have a small number of samples. Small samples are common because tasks and experimental protocols which maximally discriminate between different conditions are still under development and because of the costs associated with data collection involving human participants. For example, in our work with autistic adults, running an experiment to generate one sample of high dimensional data may require 1.5–4 hours of experimenter time (for running the experiment including set up and set down) and 3.5–6 hours of participant time (including travel time). In addition, it is difficult to recruit large numbers of autistic adults due to difficulties accessing participants and encouraging participation. Collecting samples from thousands of subjects is thus not feasible with the resources available for early stage work. However, there is still a critical need for robust and reliable machine learning (ML) methods using these smaller datasets.

High dimensional data with small number of samples are particularly common in neuroimaging studies. Arbabshirani et al. [3] conducted a survey of neuroimaging studies which used supervised ML methods to classify healthy individuals and individuals with brain disorders. Most of the surveyed studies had a small number of subjects (median 88) and interestingly, the overall reported accuracy was higher in the studies with smaller sample sizes. This effect was evident in the studies focusing on different brain disorders: schizophrenia, mild cognitive impairment, Alzheimer’s disease, depressive disorders, autism spectrum conditions, and attention-deficit hyperactivity disorder. However, Arbabshirani et al. [3] did not report statistical measures of this relationship. Varoquaux [4] also performed a meta-analysis of neuroimaging review papers which included studies focusing on various brain diseases and classification methods. Overall, similarly as in Arbabshirani et al. [3] survey, a clear pattern emerged with higher prediction accuracy reported in the studies with small sample sizes.

Despite small sample sizes being common, and the fact that limited data is problematic for pattern recognition [1, 5, 6], only a limited number of papers have systematically investigated how the ML validation process should be designed to help avoid optimistic performance estimates. Previous papers [5, 7] used synthetic Gaussian noise data to investigate how far experimental classification error is from the expected theoretical chance level. Varma and Simon [7] used a fixed sample size dataset (40 samples), and investigated the change from theoretical chance performance when using two different Cross-Validation (CV) approaches for selecting the data used for model development and model validation (Different CV methods are introduced in detail in section Validation strategies). They showed that the *nested* CV approach which avoided pooling training and testing data produced an “*almost unbiased estimate* [of performance]” [7]. In comparison, Combrisson and Jerbi [5] used only a *K-fold* CV approach and varied sample size. They found that with small sample sizes empirical accuracies overshot theoretical chance level and were more variable.

Overall, Varma and Simon [7] investigated the choice of validation method, at one fixed sample size, while Combrisson and Jerbi [5] varied sample size, but only with one validation method. In this paper we build on their work and combine the two approaches, by investigating different validation methods and systematically varying sample size. In addition, we extend the synthetic Gaussian noise data classification approach to investigate a number of additional factors influencing result reliability. Generally, the higher the ratio of features to sample size the more likely that an ML model will fit the noise in the data instead of underlying pattern [1, 6, 8]. Similarly, the higher the number of adjustable parameters the more likely the ML model is to overfit the data [9]. We quantify the effect of this by varying the feature-to-sample ratio and number of adjustable parameters in the models, as part of our synthetic data classification.

The reminder of this paper is organised as follows. First, we present a new literature review illustrating the small sample size problem. Previous reviews [3, 4] have demonstrated a negative relationship between sample size and reported classification accuracy. To show this is an ongoing issue we have performed a survey of studies which used ML algorithms in autism research, which is relatively nascent field (with only 55 studies identified for inclusion in our review). We then introduce the different validation methods in section Validation strategies. Our analysis methods are given in section Methods. We have used five clearly defined validation approaches and systematically varied sample size. We investigated classification of both Gaussian noise with an expected classification accuracy of 50% (random guessing level) and randomly generated discriminable data.

In the Results section we show that while certain validation methods produce significantly overoptimistic performance estimates (> 50%), especially when sample size is small, others are robust regardless of sample size. We also show that the feature selection process, if performed on pooled training and testing data, is contributing to bias considerably more than parameter tuning. Results for other factors apart from sample size influencing overfitting and results on different validation approaches with discriminable data are also included. After the results section we have graphically illustrated why models, developed on pooled training and testing data, can produce overoptimistic performance estimates. The same concepts as in our main simulations are exemplified in a simpler and more intuitive way, as we are aware that some readers may be less familiar with ML. Program code used for the main simulations performed in this study is provided with this article in S1 File.

## Machine learning in Autism

**Fig 1:**
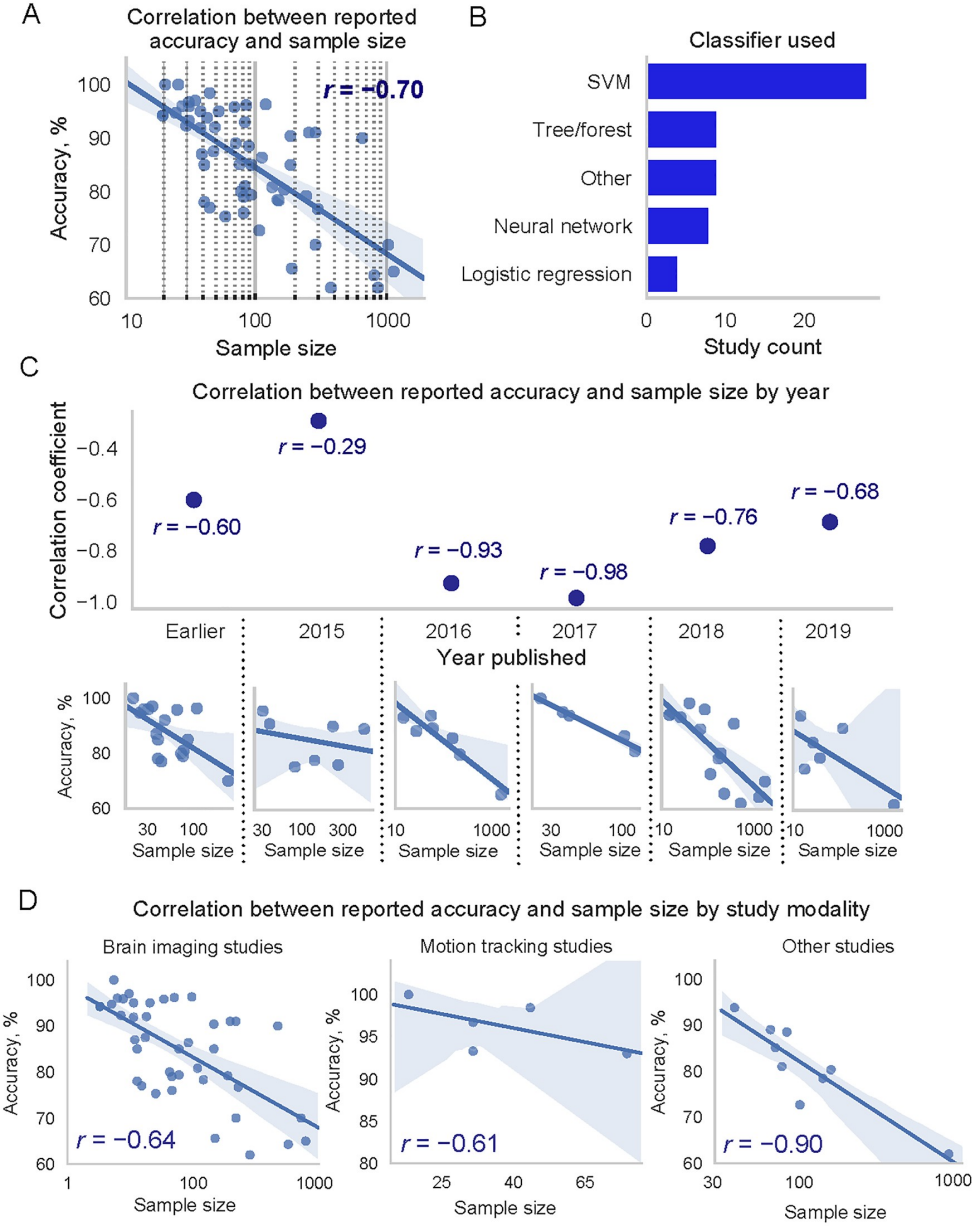
Relationship between log_10_ transformed sample size and reported accuracy. For 55 studies in the survey which applied ML methods in autism research. **A**: Relationship between log_10_ transformed sample size and accuracy, with a dark blue regression line and light blue area showing 95% confidence intervals. **B**: Classifiers used in the studies. **C**: Relationship between reported accuracy and log_10_ transformed sample size by year, bottom scatter-plots are for the studies published in that year. Year 2019 includes studies up to 18/04/2019 when the literature search was performed. N—sample size. **D**: Relationship between reported accuracy and log_10_ transformed sample size by modality of data used in the study.

To investigate the state of the art of ML in Autism research, and whether there is an effect of sample size on reported ML performance, a literature search was performed using search terms “Autism” AND “Machine learning”, detailed in Table 1. The search time period was: no start date—18 04 2019 and no search filters were used. Only studies which used ML to predict two classes and reported accuracy as a performance measure were included to ensure clear interpretation of the results. In total 55 studies were retained, with the results summarised in Fig 1. Details of the surveyed studies and measures used for analyses as well as full references are provided in S1 Table.

**Table 1:** Literature search protocol.

Search engine	Articles found	Articles retained
Web of Science	275	27
Science Direct	1018	9
Google Scholar	19600 (first 1000 viewed)	10
Other sources	24	9
Total articles retained:		55

Most of the surveyed studies had a small number of subjects (median 80). The studies used various types of data to classify autistic and non-autistic individuals, with the majority from the brain imaging domain. Other studies used, microarray, clinical chemistry, cognitive, motion and eye tracking data. Studies also used different data pre-processing, feature selection and classification methods, Fig 1B. In our survey We explored if even after combining such varied studies there was a relationship between sample size and accuracy. A Kolmogorov-Smirnov test indicated that sample size in our surveyed studies did not follow a normal distribution, *D*(55) = 0.28, *p* < 0.001. A distribution was strongly positively skewed and leptokurtic because of high proportion of small sample studies. We have applied log_10_ transformation to sample size data to resolve this issue. After the transformation, the data was normally distributed, *D*(55) = 0.12, *p* = 0.06, and we found a strong negative relationship between log_10_ transformed sample size and reported accuracy, *r*(53) = −0.70, *p* < 0.001. By not transforming the data and using non-parametric Spearman’s rank-order correlation we found a correlation of a similar magnitude, *r*(53) = −0.67, *p* < 0.001, but we chose to transform sample size data for clearer graphical representation in Fig 1. Forty nine percent (*R*^2^ = 0.49) of variance in reported accuracy was simply explained by the sample size. Examining the relationship between reported accuracy and log_10_ transformed sample size by year a consistent negative relationship was evident, Fig 1C.

We have also performed additional analyses to exclude the possibility that the negative relationship between sample size and reported accuracy was dependent on the data modality. The survey was dominated by brain imaging studies and only one other modality of motion tracking contained more than two studies allowing separate correlation analyses. We have combined the rest of the studies into a category: *other*. The results show that a negative relationship between sample size and reported accuracy was evident in different modalities, Fig 1D. The correlation between log_10_ transformed sample size and reported accuracy was *r*(39) = −0.64, *p* < 0.001, for brain imaging studies, *r*(3) = −0.61, *p* < 0.271 for motion tracking studies, and *r*(7) = −0.90, *p* = 0.001 for other studies, suggesting that the negative relationship between sample size and reported accuracy was independent of the study modality.

A strong relationship between sample size and reported performance suggests that ML models are biased to produce overoptimistic results when a sample used to train them is small Fig 1. In supervised learning, the ideal model would both approximate the regularities in the training data, and would generalise to unseen new data. However, this is unlikely because training data may include noise and may not represent the population sufficiently well. Too complex models are likely to represent noise in the training data, rather than underlying patterns of interest. Such models overfit the training data. In contrast, too simple models are likely to underfit training data and fail to capture underlying regularities. Obviously, one would aim to construct a model which fits training data just enough to capture a pattern which is representative of the population, but does not fit the noise inherent in the available training data. Underfitting is improved by simply applying models of increasing complexity, however, overfitting, is a more difficult problem. To assess and control overfitting, model validation is commonly used.

## Validation strategies

**Fig 2:**
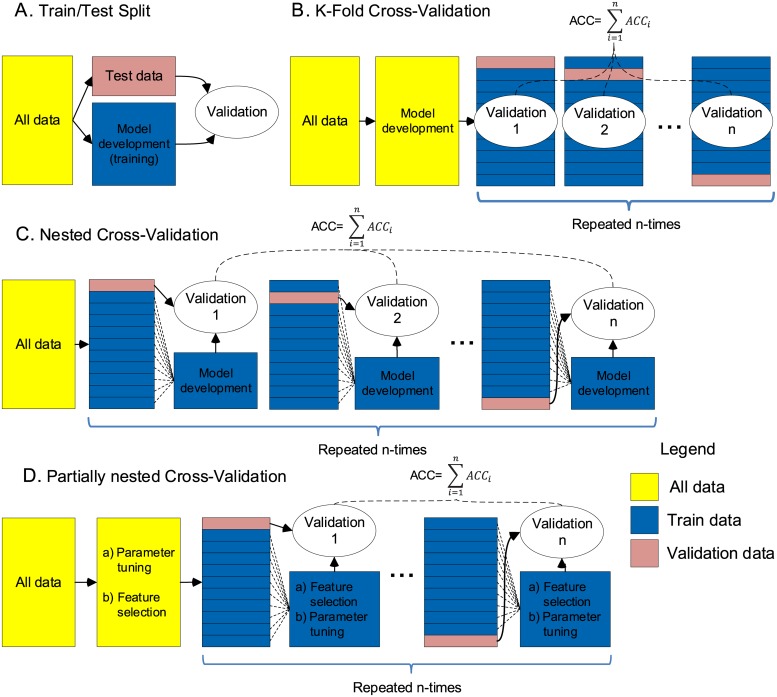
Validation methods. **A**: Train/Test Split. **B**: K-Fold CV. **C**: Nested CV. **D**: Partially nested CV. ACC—overall accuracy of the model, *ACC*_*i*_.—accuracy in a single CV fold.

A reliable way to validate a ML model’s performance is to train a model with available data and assess its classification performance using newly collected data or a separate dataset. Another reliable approach, commonly called Train/Test Split, is to separate a portion of data before developing a ML model and to use that data only for validation, Fig 2A. Using *unseen* data to test a ML model gives an unbiased estimate of what performance would be when the model is deployed for actual predictions in real-world situations. However, these approaches require one to collect or hold a substantial amount of data for validation, and are rarely used in research involving human participants, where data collection is commonly associated with high costs.

Cross-Validation is a common solution when the available datasets are limited. Instead of training a fixed model only once as in Train/Test split, iteratively several models are developed on different portions of the data. K-Fold is a common CV approach. First, a well-defined model is developed by normalising data, selecting features, tuning parameters and/or performing other development steps. Then a portion of data is separated for validation leaving the rest to train a model and predict the classes on the left-out validation data. This process is repeated several times, by leaving out a different portion of the data for validation until all the data is used. The model’s performance is then calculated as a mean of classification performances, in each of the validation folds, Fig 2B.

When validation with a separate dataset is not feasible because of small sample size, K-Fold CV is very economical as it allows one to use all the data for training and also to reuse all of it for validation. If validation was to be performed with a separate dataset, double the amount of data would be needed to have the same quantity of data for training and validation. More importantly CV theoretically should give more accurate out of sample error estimation, compared to previously discussed approaches. Using all of the available data for validation should give out of sample error estimates which are less influenced by noise and are more representative of the population, compared to using a portion of the data as with Train/Test Split or new dataset.

However, K-Fold CV does not ensure that the data used to validate the classifier is not part of the data used to train it. Stone [10] pointed out the importance of separating CV used for model development and CV used for model evaluation. Varma and Simon [7] have demonstrated that using the data to validate a model which was also used to develop it can produce overoptimistic performance estimates.

One possible solution which avoids pooling training and validation data, but at the same time is economical (allows all the data to be used for training and reuse for validation) is Nested CV [7, 11], Fig 2C. A portion of data is split at the beginning and in each CV fold a model is then developed on the reduced training set from scratch, including feature selection and parameter tuning. This is repeated with splitting a different portion of the data for validation, and each time developing a new model for training until all the data is used. Varma and Simon [7] suggest that Nested CV provides almost unbiased performance estimates.

## Methods

In this study, by using Gaussian noise as data, we have simulated a situation in which robust validation should produce two-class classification accuracy approaching theoretical chance level of 50%. We tested five validation approaches: Train/Test Split, K-fold, Nested, and two types of partially nested CV. Importantly, we performed these simulations using different sample sizes to provide an insight into whether the tendency to report higher performance estimates with smaller sample size could be due to insufficiently reliable validation. In addition, we have tested what other factors, apart from sample size, influence overfitting and how different validation methods perform with discriminable data. To show that simulation results generalise to algorithms differing in complexity, two algorithms were used. One, computationally demanding and complex, where Support Vector Machine (SVM) [12] classifier with Radial Basis Function (RBF) kernel was coupled with Support Vector Machine Recursive Feature Elimination (SVM-RFE) [13] feature selection. Another, simpler, where the logistic regression classifier was coupled with two-sample *t*-test feature selection.

### Overview of procedures

Typically, ML algorithm development starts with data cleaning and outlier removal, then the data is normalised to ensure that separate features have a balanced influence on the labels. Then if number of features is large, which is especially true for neuroimaging and gene expression studies [3, 14, 15], feature selection is performed. This is done because ML algorithms tend to achieve optimal performance in a reduced feature space [6, 16]. Many of the ML models include hyper-parameters which can be fine-tuned. This process is commonly coupled with CV to not only achieve optimal algorithm performance, but also to control overfitting. Finally, the model is validated to ensure that it generalises to “unseen” data. Below the development stages of ML algorithms which were used in this study are described with a particular emphasis on validation.

### Data

Data was simulated by randomly drawing values from a Gaussian distribution with a mean of zero and standard deviation (SD) of one. Numpy (Python) method random.normal was used to generate pseudo-random values drawn from standard normal distribution. Binomial classification was used, and each simulated dataset was split into two equally sized subsets for each class. Because features did not bear meaningful relationship to labels, and classes were balanced, binomial classification models would be expected to yield chance level classification accuracy of 50%.

### Data normalisation, cleaning

As the data was drawn from standard normal distribution data normalisation was not necessary and was omitted. Data cleaning was also not necessary in this case; however, with real datasets, missing value replacement/removal and outlier replacement/removal are usually necessary steps.

### Classification algorithms

For classification, SVM [12] was chosen because it was by far most commonly used algorithm in our survey, Fig 1B. We have also used a logistic regression algorithm.

*SVM* separates the classes by maximising the gap between training examples from each class. The examples in the test data are when assigned a label based on which side of the gap they fall. The SVM algorithm assumes linear separability of classes, however in reality this assumption in rarely realistic. Therefore, a regularisation parameter *C* is introduced which weighs the importance of misclassification and allows SVM to fit a linear separating hyperplane with some of the examples being misclassified. Another method to deal with non-linearly separable classes is to use kernel functions. Kernel functions project features to a higher dimensional space. This enables the separation of classes which are non-linearly separable in the original space with a linear hyperplane in a higher dimensional space. In this study SVM with RBF kernel was used. RBF kernel has a regularisation parameter *γ*, which regulates the spread of the kernel function and in turn determines the flexibility of the separating hyperplane. SVM was implemented with Libsvm library [17].

*A logistic regression* model was also used as a classifier. It uses logistic function to predict binary classes based on linear combinations between class and features. Logistic regression was implemented using Scikit-learn library [18].

### Parameter tuning of classification algorithms

SVM-RBF regularization parameters *C* and *γ* were optimized to improve classification and to control overfitting. Both parameters regulate the complexity of a separating hyperplane. By setting a low penalty for misclassification (parameter *C*) SVM tries to classify all training examples correctly making a separating boundary complex. Similarly, the higher the value of kernel’s *γ* parameter, the more flexible and complex the separating boundary. To optimise *C* and *γ* parameters we used a grid search approach, which evaluates classification accuracy with different combinations of *C* and *γ* parameters by using 10-fold CV. A pairing of *C* and *γ* parameters which gave the highest CV accuracy was selected. Grid search was performed with parameters set to: *C* = 2^*j*^, where *j* = 1, 2, … 7 and *γ* = 2^*i*^, where *i* = -1, -2, … -7. Grid search was implemented with Scikit-learn library. For visualisation of how parameters influence SVM decision boundary see Devos et al. [19]

To control for overfitting logistic regression was regularised using *L*_1_ (Lasso) or *L*_2_ (Ridge) penalty terms and the magnitude of penalty was controlled by regularization parameter *C*. Like in SVM, smaller values of *C* specify stronger regularization. To optimize parameters grid search was performed with penalty set to *L*_1_ or *L*_2_ and *C* set to *C* = *e*^*i*^, where *i* = 0, 1, … 9.

### Feature selection

Most of the studies in our own and other surveys [3, 4] used feature selection. Therefore, for our simulations we initially generated 50 features comprised of Gaussian noise and used feature selection to reduce dimensionality. We chose two feature selection methods, one computationally complex—SVM-RFE, and another simpler—two-sample *t*-test.

*SVM-RFE* algorithm selects features based on how important they are for an SVM classifier to separate classes. SVM-RFE starts with a full feature set and in a number of iterations eliminates a set number of features which are deemed least important for separating classes by an SVM algorithm, using weight vector of dimension length(s) as a ranking criterion [13]. The algorithm removes least important features in iterations because in each iteration the relationship between features and labels changes. Top-ranked features are not necessarily the most relevant individually; they are, however, optimized by considering interdependencies with other features and the class. The final feature set is selected from the iteration in which SVM achieves best classification performance. In this study a single feature was eliminated in each SVM-RFE iteration and the final feature set was selected based on the highest classification accuracy by linear SVM with *C* set to 1.

*Two-sample t-test* was also used for feature selection. In contrast to SVM-RFE it is a much simpler method which ranks features based on how different feature means are between the two classes. In this study, 10 features with the highest absolute value of *t* statistic were selected.

### Validation and performance evaluation

For validation of the results, five different validation approaches were used and their performance was compared.

*Train/Test Split* is a simple and reliable validation approach. A portion of the data was split before any model development steps and it was used only once to validate the developed model Fig 2A.

*K-Fold CV*. First, a single well-defined model was developed by selecting features and tuning parameters, Fig 2B. Then the model was validated by separating one-tenth of the data for validation and the rest for training. CV process was iteratively repeated ten times. In each fold, a different one-tenth of the data was selected for validation. In such way, in the end, all the data was used for training and also for validation. The final performance of the model was then calculated as a mean of classification performances in each of the ten validation folds.

*Nested CV* is performed in two layers to achieve training and validation separation, Fig 2C. In this study, ten-fold Nested CV was used. In the outer layer, 10% of the data was separated for validation and the rest of the data was used to develop a model. In the internal layer, the remaining 90% of the data was used for feature selection and parameter tuning. A developed model was then validated with 10% of the data which was split at the beginning. This process was repeated 10 times by selecting a different 10% of the data for validation and by using a different 90% of the data to develop a new model from scratch. The overall performance was then calculated as a mean of classification performances of the 10 separately developed models on different 10% sets of the validation data which was not involved in developing the models.

*Partially nested validation*. Arbabshirani et al [3] in their large survey noted that in most studies, feature selection was performed in a non-nested fashion. Feature extraction/selection can be computationally very demanding which could explain why it is frequently performed only once on pooled training and testing data, instead of performing it in each CV fold. In this study, to examine whether accuracy estimates are biased more by feature selection or parameter tuning we performed two types of partially nested validation, Fig 2D. First, feature selection was performed in a non-nested fashion and parameter tuning in nested fashion. That is, feature selection was performed once in an outer nested validation loop, on pooled training and validation data. Only parameter tuning was nested and performed 10 times, avoiding the pooling of training and validation data. Second, feature selection was performed in nested and parameter tuning in a non-nested fashion.

### Implementation

The results section is organised as follows. First, we compare five different validation methods using Gaussian noise data as features. Data was split into two equally sized subsets for each class. The feature set started with 50 features and was reduced by using feature selection. Sample size was manipulated, and classification results were evaluated using Train/Test Split, K-fold CV, Nested CV and two types of partially nested CV. The performance estimate was accuracy. To obtain accuracy distributions models were trained 50 times at each sample point, then validation results were compared against theoretical chance level using confidence level of 95%.

Then, other factors apart from sample size which can lead to overfitting with K-fold CV were investigated. We kept sample size constant, but manipulated number of Gaussian noise features. As feature-to-sample ratio is well known to influence classification result reliability [20–22], we additionally manipulated feature-to-sample ratio from 1/3 to 3 and show that feature/sample size ratio can be a good indicator of how much a model is likely to overfit. We also investigated how overfitting was influenced by grid size used to fine-tune classifier hyper-parameters and number of CV folds.

Finally, different validation methods were compared by using discriminable data to investigate an interaction between the increase in classification ability [23–25] and the reduction in overfitting with larger samples. Discriminable datasets were generated with 50 features and balanced labels. 40 of the features were generated from zero-mean and one SD Gaussian noise for both classes. To create discriminability, the remaining 10 features for the first class were generated from Gaussian noise with a mean of 0.5 and SD of one while for the second class from the Gaussian noise with a mean of 0 and SD of one.

Unless indicated differently all model parameters were set as described in this section.

## Results

### Comparison of different validation methods

**Fig 3:**
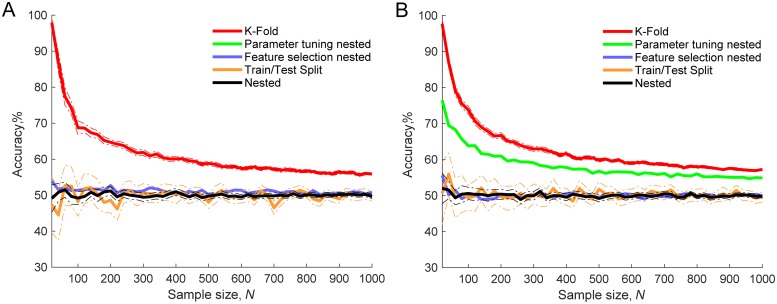
Gaussian noise classification accuracy distributions with different validation approaches. K-Fold, Nested, Train/Test Split and two types of partially nested validation methods used. Thick lines show mean validation accuracy and dash-dot lines show 95% confidence intervals for 50 runs. **A**: SVM-RFE feature selection and SVM classification. **B**: *t*-test feature selection and logistic regression classification.

The effect of sample size on how close the empirical classification result is to theoretical chance level was examined. The sample size was manipulated and ranged from 20 to 1000. To evaluate classification results K-Fold, Nested, Train/Test Split and two types of partially nested validation were used. Fig 3 shows that by using both a complex algorithm, where SVM-RFE feature selection was coupled with a SVM-RBF classifier (SVM algorithm hereafter, Fig 3A), and a simpler algorithm, with *t*-test feature selection and a logistic regression classifier (logistic regression algorithm hereafter, Fig 3B), accuracies given by K-Fold CV were considerably higher than the theoretical chance level of 50%. The highest difference was observed with smaller sample sizes; however, the difference was still evident even at the sample size of *N* = 1000. In contrast accuracy distributions produced by using Nested CV and Train/Test Split did not statistically significantly differ from 50% chance level with SVM and logistic regression algorithms at 96.5% sample size points (*p* ranged from 4.3 × 10^−4^ to 0.997, a small number of significant differences is expected by chance with 95% confidence level).

Two types of partially nested validation were also performed. In the first instance, only parameter tuning was nested while feature selection was performed on the pooled training and testing data in non-nested fashion. Fig 3 shows that nesting parameter tuning only was not sufficient to control overfitting. By using both SVM and logistic regression algorithms, empirical accuracies at each sample point were significantly higher than the 50% chance level (*p* ranged from 1.1 × 10^−42^ to 6.3 × 10^−20^ and from 1.7 × 10^−33^ to 1.7 × 10^−22^ respectively). The results were considerably different when feature selection was nested and only parameter tuning was performed on the pooled training and testing data. The curves approached 50% chance level, Fig 3. One sample *t*-tests showed that for the SVM algorithm empirical accuracy distributions were significantly higher than 50% chance level on 56% sample size points (*p* ranged from 2.8 × 10^−5^ to 0.879). For a logistic regression algorithm accuracy distribution using this type of partially nested validation was higher than the chance level only at one sample size point (2%, *p* ranged from 0.039 to 1.0).

Taken together, partially nested validation results show that to perform feature selection in nested fashion is paramount for controlling overfitting, while nesting parameter tuning has a smaller effect. This was the case for our data and models used. However, for other situations, especially, if feature selection is not used or relied less, parameter tuning could contribute to overfitting more. Our models relied on feature selection substantially, reducing feature number from 50 to 10, to represent ML studies for disorder prediction, which commonly use feature selection as an important model development step [3, 26].

### Other factors apart from sample size influencing overfitting

**Fig 4:**
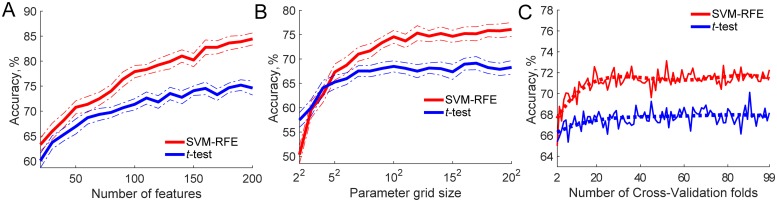
Other factors apart from sample size influencing overfitting when K-Fold CV is used. SVM-RFE and *t*-test feature selection, SVM classification, and sample size fixed at *N* = 100. **A**: Feature number manipulated from 20 to 200. **B**: Parameter tuning grid size manipulated from 2 × 2 to 20 × 20 with *C* = 2^*j*^, where *j* varied from 2 to 20 and *γ* = 2^*i*^, where *i* varied from −2 to −20. **C**: Number of CV folds varied from two-fold to leave-one out. Thick dashed lines show fitted 5^*th*^ order polynomial trend.

**Fig 5:**
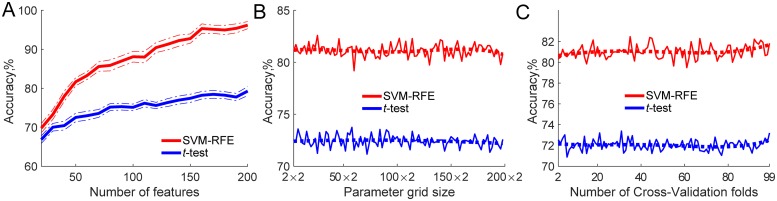
Other factors apart from sample size influencing overfitting when K-Fold CV is used. SVM-RFE and *t*-test feature selection, logistic regression classification, and sample size fixed at *N* = 100. **A**: Feature number manipulated from 20 to 200. **B**: Parameter tuning grid size manipulated from 2 × 2 to 200 × 2 with penalty set to *L*_1_, *L*_2_ and *C* = *e*^*i*^, where *i* varied from −4 to 4. Thick lines show fitted 5th order polynomial trend. **C**: Number of CV folds varied from two-fold to leave-one-out. Thick dashed lines show fitted 5^*th*^ order polynomial trend.

Other factors influencing overfitting with K-Fold CV were examined. Sample size was kept constant at *N* = 100 while feature number, parameter tuning grid size and number of CV folds were manipulated. Both SVM-RFE and *t*-test feature selection were used in combination with SVM-RBF classifier (Fig 4) and logistic regression classifier (Fig 5).

#### Number of features

For both SVM (Fig 4A) and logistic regression (Fig 5A) classifiers feature number had a clear influence on overfitting. The higher the number of features used, the greater the difference was between the empirical classification accuracy and theoretical chance level of 50%. There was also a clear difference between the feature selectors used. SVM-RFE accuracies were higher than *t*-test, and this difference became greater with increasing feature space to select from.

#### Classifier hyper-parameter optimisation

Grid size used to fine-tune SVM-RBF *C* and *γ* parameters also had a clear influence on overfitting, as grid size increased, achieved accuracies also increased (Fig 4B). There was, however, no such effect on accuracy when the grid size to fine-tune logistic regression parameters was increased (Fig 5B).

#### Number of CV folds

Similar results were observed when the number of CV folds used to fine-tune the model’s hyper-parameters was increased. With SVM-RBF classifier, the use of a higher number of folds affected accuracy up to approximately 20 folds when the effect levelled off. There was again no clear effect when the number of CV folds was increased with the logistic regression classifier.

### Feature-to-sample ratio

**Fig 6:**
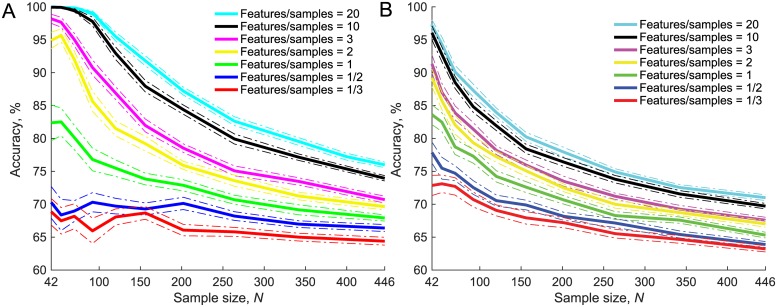
K-Fold CV with different feature-to-sample ratios. Sample size ranged from 14 to 446 and feature number was set accordingly to keep feature-to-sample ratios at 20, 10, 3, 2, 1, 1/2 and 1/3. **A**: SVM-RFE feature selection and SVM classifier. **B**: *t*-test feature selection and logistic regression classifier.

The results on other factors influencing overfitting showed that the number of features used to develop a model had a clear impact on overfitting regardless of the feature selector or classifier used. This effect was investigated further as it is likely that feature-to-sample ratio could be a better indicator of how much a model is likely to overfit compared to sample size alone. Sample size was manipulated from 42 to 446 and feature number was also manipulated accordingly, to achieve different feature-to-sample ratios of 1/3, 1/2, 1, 2, 3, 10 and 20, Fig 6. The results showed that feature-to-sample ratio is a good indicator of how much a model is likely overfit. By using both SVM (Fig 6A) and logistic regression (Fig 6B) algorithms the accuracies achieved by models with higher feature-to-sample ratios were greater.

### Validation with discriminable data

**Fig 7:**
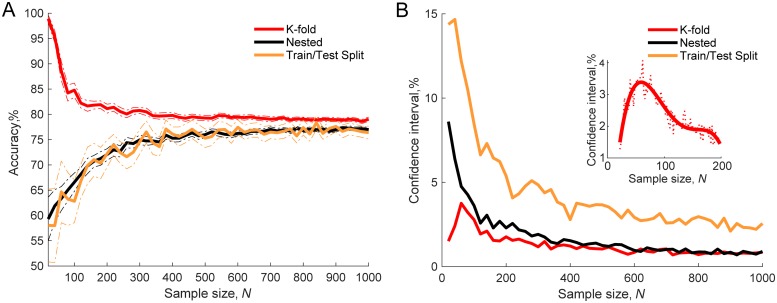
Classification with discriminable data using K-Fold CV, Nested CV and Train/Test Split validation methods. **A**: Comparison of different validation methods. Dash-dot lines show 95% confidence intervals for 50 runs. **B**: Size of 95% confidence intervals. Inset plot shows more refined view of confidence intervals for K-fold CV in a sample size range of 20 to 200 (in the inset plot sample sizes were *N* = 20, 22, … 198, 200, in the main plot *N* = 20, 40, … 980, 1000).

To make data discriminable, 10 out of 50 features were generated from Gaussian noise with means differing between classes (see section Implementation). SVM-RFE was used for feature selection and SVM-RBF for classification. Models were validated using different validation methods: K-Fold CV, Nested CV and Train/Test Split. Fig 7A shows that performance estimates were varied. There were no significant differences in accuracy between Train/Test Split validation and Nested CV at 96% of sample size points, two-sample *t*-test (*p* ranged from 0.039 to 0.995). K-fold CV however gave significantly higher performance estimates compared to Nested CV (*p* ranged from 1.3 × 10^−6^ to 5.4 × 10^−35^) or Train/Test Split validation (*p* ranged from 3.8 × 10^−1^ to 5.3 × 10^−19^). The accuracy for both Train/Test split and Nested CV increased with sample size and then levelled off when sample size reached approximately *N* = 700 at ≈ 77% accuracy level. This shows a well-known aspect of ML models; with a larger training sample size, models have higher statistical power to learn a pattern discriminating between classes and achieve higher performance. Several studies have explored this by calculating learning curves which show accuracy as a function of the training sample size [23–25] and assume a typical shape similar to Nested CV or Train/Test Split curves in Fig 7A. The K-fold CV curve, on the other hand, was not of the typical learning curve shape. Although an increase in learning must have been present with larger sample sizes, the overfitting had a stronger effect. This was further shown by exploring variability of performance estimates expressed as 95% confidence intervals, Fig 7B. Similarly, as in [4, 5] we found that with larger sample sizes variability in performance estimates decreased. This was not the case for K-fold validation where variability increased up to *N* ≈ 60 and then started decreasing, Fig 7B inset. This was caused by a high frequency of perfect classification (100%) occurrences at sample sizes below 60.

## Illustrative examples of why models overfit

Our simulations show that validating models with the data which was also involved in model training can lead to overfitting and overoptimistic performance estimates. However, it may not necessarily be intuitive why overfitting occurs. Here we graphically illustrate how two model development stages, parameter tuning and feature selection, can lead to overfitting if those development stages are performed on the pooled training and validation data. The examples are small and simple, illustrating the concepts explored in our main simulations in a more intuitive way.

### Parameter tuning and overfitting

**Fig 8:**
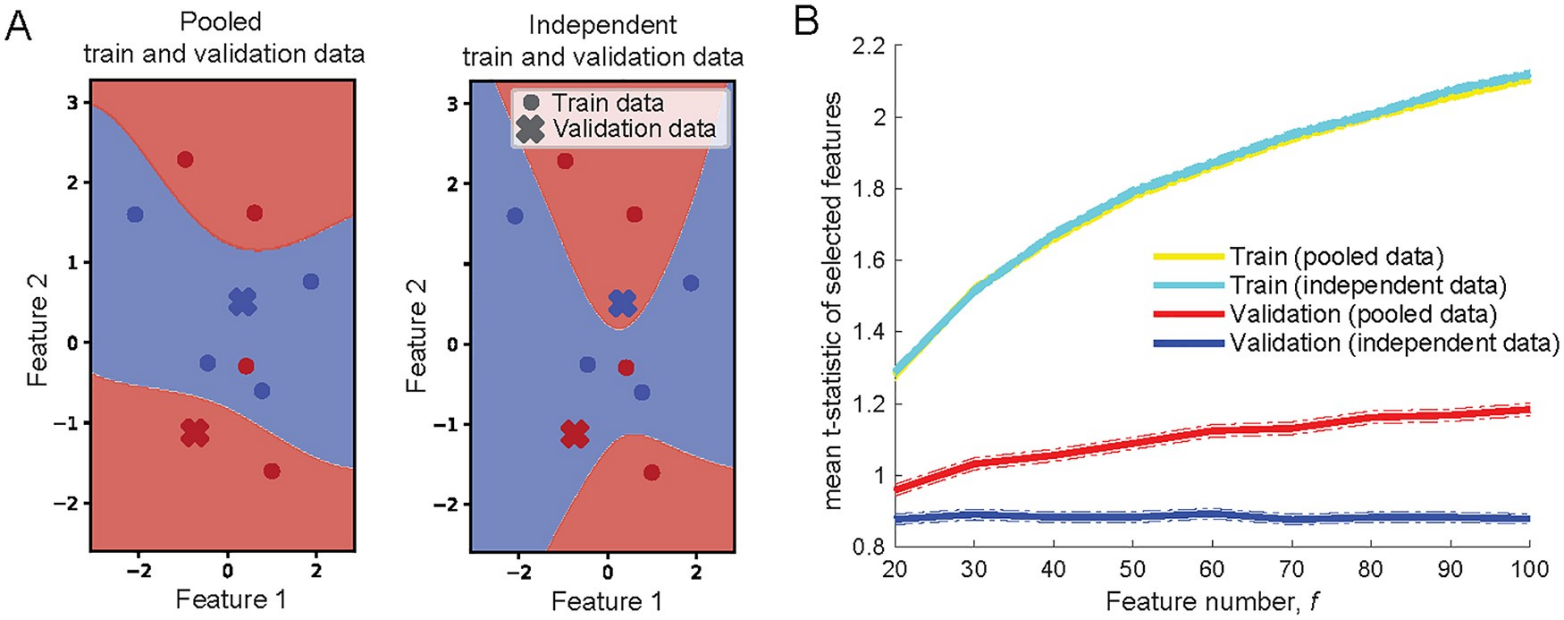
Illustrative examples of why models overfit. **A**: SVM-RBF decision boundary. Red and blue circles/crosses show data points from two classes, red and blue areas show learned decision boundary by SVM-RBF. **Left**: Classifier trained on both train data points (circles) and validation data points (crosses). **Right**: Classifier trained only on train data points (circles). **B**: Two-sample *t*-test feature selection performed both, on pooled and on independent train and validation data. Y axis shows mean *t*-statistic for selected 10 features from the pool of features ranging from 20 to 100.

To illustrate how parameter tuning can lead to overfitting we have used a small classification example with a sample size of 10 (5 samples from one class and 5 samples from a second class) and only two features. The data was generated from random Gaussian noise and SVM-RBF (with the same settings as in the main analyses) was used to separate data points from two classes (shown in red and blue in Fig 8A).

We have developed two models in parallel on the same data and with the identical settings. To validate we have used the same two data points for both models. The only difference between the models was that the first model (Fig 8A left) was trained on all 10 data points—on the pooled training and validation data. The second model (Fig 8A right) was developed by keeping training and validation data independent. It was trained on 8 data points making the remaining 2 validation data points ‘’invisible’’ to the model. Decision boundaries (lines separating blue and red areas in Fig 8A) were clearly differently *learned* by the two models. The first model fitted the data which was used for validation and classified two validation data points correctly, while the second model, for which validation data was ‘invisible’ misclassified the same two validation data points.

By running these two models 1000 times, each time with different randomly generated data, both models were capable of fitting training data and achieve over 80% classification accuracy, showing that the models were capable of fitting random noise well. The first model, however, also fitted noise in the validation data achieving mean accuracy of 81%, while the second model for which validation points were ‘invisible’ achieved accuracy rate of 50%, random guessing level.

Overall, this example graphically illustrates that complex enough models are capable of fitting random noise in the data. It also shows that if the data, which is used for validation, is also involved in parameter tuning, the performance is inflated because the models fit the noise not only in training but also in validation data.

### Feature selection and overfitting

To graphically illustrate how performing feature selection on pooled training and validation data can lead to overfitting we have used *t*-test feature selection. *t* statistic of two-sample *t*-test simply shows how different are the means between two classes in units of standard error. We have used Gaussian noise data of 50 samples equally balanced between two classes and performed *t*-test feature selection to select 10 features from sets varying from 20 to 100 features.

20% of the data was randomly split for validation and once again we have performed feature selection using two different approaches. in the first instance it was performed on the pooled training and validation data and in the second instance only training data was used for feature selection. This was repeated 100 times.

Fig 8B shows that with the larger pool of features to select from, the selected ten features had greater between-class mean differences than with the smaller pool of features to select from (train lines in Fig 8B). This was the case for both approaches. The main difference between two approaches was in validation. Selecting 10 features with greatest mean differences on pooled training and validation data (100% of data) also led to greater between-class mean differences in validation data alone (randomly split 20% of data). The effect also increased with larger feature pool to select from. This was not the case when validation data was independent of feature selection. Despite mean differences being high in ten selected features in the training data (80% of data), the selected features produced low mean differences on validation data (20% of randomly split data) which would be expected by chance.

This example shows that feature selection is capable to select features as discriminative between classes because of inherent noise (in this example the data was Gaussian noise with between class differences occurring by chance). It also shows that if feature selection is performed on pooled training and validation data, the validation data can occur discriminative because of noise.

## Discussion

Robust evaluation of ML classification is imperative for ML research as it allows meaningful comparisons between different studies and different methods. Robust evaluation is even more important when available training and testing samples are small [1, 6].

Our results demonstrate the importance of separating training and testing data to avoid optimistically biased performance estimates. K-Fold CV was not sufficient to control overfitting. By simply testing the performance of the algorithm with the data which was also involved in algorithm training was enough to produce biased results with small sample sizes. However, a substantial bias still remained even with sample size of 1000. On the other hand, similar to [7] we have found that Nested CV gave unbiased performance estimates. Furthermore, Nested CV results were unbiased regardless of the sample size.

Additionally, we examined which model development stage, feature selection or parameter tuning is implicated in validation bias more. Partially-nested validation results showed that by performing only feature selection in a non-nested fashion gave considerably biased results, while the bias was much smaller when only parameter tuning was performed in non-nested fashion. In many studies, the initial number of measures (features) can be very large. In their survey Arbabshirani et al. [3] noted that most of the neuroimaging studies consisted of two parts. In the first part using statistical tests, such as *t*-tests, differences between groups were identified. In the second part features, which were preselected in the first part, were used for classification. As a result, feature selection was performed on the pooled training and testing data and posed a bias. Although, performing feature selection multiple times in nested fashion with high dimensional data can be computationally demanding, our simulations have shown that it is necessary to avoid overfitting. Additionally, other model development stages which were not examined in this study (e.g. normalisation, outlier removal) if performed on pooled training and testing data could also lead to biased results.

We have also investigated other factors which could influence overfitting when K-Fold CV is used. Our results have demonstrated that feature-to-sample ratio is a good indicator of how much a model is likely to overfit. The accuracies achieved by models with higher feature-to-sample ratios were greater. Increasing the set of parameters over which a model is optimized also increased amount of bias with SVM model. There was no such effect with logistic regression. Similarly, greater number of CV folds used for parameter tuning had only a slight effect with SVM model and no effect with logistic regression model.

Our simulations with discriminable data showed that by using Nested CV and Train/Test split validation, models were capable of learning a pattern in the data more efficiently and achieved higher performance with larger sample sizes. This is consistent with previous studies which investigated accuracy as a function of the training sample size [23–25]. Interestingly, the distance between K-fold and Nested CV curves with non-discriminable data (Fig 3A) was larger than with discriminable data (Fig 7A) at each sample size point. This suggests that with more discriminable data bias produced by less robust validation is lower and also the opposite, the less discriminable the data the higher the importance of robust validation.

Validation results were highly variable as can be seen from 95% confidence intervals included in each graph. The same algorithm used on the data drawn from the same distribution produced broad range of performance estimates. The variability of performance estimates was also greater with smaller sample sizes, Fig 7B. In most studies in our survey, a single performance estimate was reported. However, as our results indicate and as noted by Varoquaux [4] intrinsically large sampling noise signifies the importance of reporting confidence intervals. Practically a distribution of performance measures could be produced by, for example, multiple times differently/randomly splitting the data to CV folds [27].

Only a few previous studies systematically investigated ML validation caveats associated with small sample size. Arbabshirani et al. [3] and Varoquaux [4] reviewed studies, which applied ML methods for prediction of various brain disorders and found that studies with smaller sample sizes tended to report higher classification performance. In this paper, as an illustrative example we have performed a survey of studies which used ML classification to predict autism or absence of autism (two class problem) and indeed found a significant negative relationship between sample size and accuracy, *r*(53) = −0.70, *p* < 0.001. Study surveys by Arbabshirani et al. [3], Varoquaux [4] and our survey, all suggest the importance of robust application of ML methods. To help in this process, there are good guideline studies advising how to avoid pitfalls, including how to reliably validate the results [28, 29].

Like in our simulations Combrisson and Jerbi [5] used Gaussian noise data to investigate how much empirical classification performance differed from theoretical chance level. Several classifiers coupled with K-fold CV were used. Empirical accuracies overshot theoretical chance level and were more variable when sample sizes were small. The researchers interpreted this discrepancy between theoretical and empirical accuracy level as arising from the fact that small samples give a bad approximation of true randomness. Our simulations, however, show that this is not the case. In contrast to Combrisson and Jerbi [5] in our simulation we used not only K-fold CV but also other validation methods. Nested and Train/Test Split validation results showed no deviation from the theoretical chance level regardless of the sample size and the effect of bad approximation of true randomness was not present. Overoptimistic performance was observed only with validation methods in which the validation process pooled training and testing data.

Varma and Simon [7] also used synthetic Gaussian noise data to investigate how far experimental two-class classification error was from expected 50% theoretical error. The researchers found that by using K-fold CV mean error was considerably overoptimistic. On the other hand, Nested CV provided almost unbiased performance estimates. The researchers, however did not investigate the influence of sample size, which for all simulations was fixed at *N* = 40. We show that not only with *N* = 40 but even at larger sample sizes of up to *N* = 1000 K-fold CV bias remains, although it becomes less prominent with larger sample sizes.

Combrisson and Jerbi [5] have shown that K-fold CV is more biased with small sample size, while Varma and Simon [7] have shown that K-fold CV is biased, and Nested CV is not at a fixed sample size. We have filled the gap by investigating both factors associated with bias, namely validation method and sample size together. We have demonstrated that validation methods which do not separate training and testing data at model development stage lead to overoptimistic performance estimates. Moreover, the bias is strongest with small sample sizes. This gives a good indication why in our own and other surveys [3, 4] there was a negative relationship between reported performance estimates and sample size.

## Conclusion

We show that K-Fold CV provides optimistically biased performance estimates and is not sufficient to control overfitting. Testing algorithm performance on the data which was used for training the same algorithm is enough to produce strongly optimistically biased results when a sample size is small. However, a substantial bias remains even with respectable sample sizes. In contrast, Nested CV provides unbiased performance estimates. Completely separating testing and training data is enough to obtain unbiased performance estimates regardless of sample size. When only part of model development is performed in a nested fashion, nesting feature selection is more important than parameter tuning to avoid overfitting. Other factors which influence bias, such as data dimensionality, hyper-parameter space, number of CV folds and data discriminability should also be considered.

## Supporting information

S1 File A supporting data file in .py format containing Python code used for simulations. (PY)

S1 Table A table of studies included in the study survey. (PDF)
