# Supplementary material for: Machine learning algorithm validation with a limited sample size
Source: PLoS One. 2019 Nov 7;14(11):e0224365. doi: 10.1371/journal.pone.0224365 (PMC6837442; doi:10.1371/journal.pone.0224365)
Supplement: S1 Table — (PDF) [file pone.0224365.s002.pdf]

Supporting information: S1 File. Summary of studies included in the study survey.

Study	Modality	Classifier	Sample size	Accuracy, %
Akshoomoff et al. 2004 [1]	Brain imaging	Discriminant function analysis	67	95.8
Jiao et al. 2010 [2]	Brain imaging	Tree/forest	38	87.0
Ecker et al. 2010 [3]	Brain imaging	SVM	44	77.0
Ecker et al. 2010 [4]	Brain imaging	SVM	40	85.0
Ingalhalikar et al. 2011 [5]	Brain imaging	SVM	75	80.0
Anderson et al. 2011 [6]	Brain imaging	Thresholding	80	79.0
Uddin et al. 2011 [7]	Brain imaging	SVM	48	92.0
Murdaugh et al. 2012 [8]	Brain imaging	Logistic regression	27	96.0
Uddin et al. 2013 [9]	Brain imaging	Logistic regression	40	78.0
Deshpande et al. 2013 [10]	Brain imaging	SVM	30	95.9
Just et al. 2014 [11]	Brain imaging	Naïve Bayes	34	97.0
Wee et al. 2014 [12]	Brain imaging	SVM	117	96.3
Segovia et al. 2014 [13]	Brain imaging	SVM	92	85.0
Zhou et al. 2014 [14]	Brain imaging	Tree/forest	280	70.0
Jamal et al. 2014 [15]	Brain imaging	SVM	24	94.7
Chen et al. 2015 [16]	Brain imaging	Tree/forest	252	91.0
Jin et al. 2015 [17]	Brain imaging	SVM	80	76.0
Plitt et al. 2015 [18]	Brain imaging	SVM	296	76.7
Iidaka et al. 2015 [19]	Brain imaging	Neural network	640	90.0
Libero et al. 2015 [20]	Brain imaging	Tree/forest	37	91.9
Li et al. 2016 [21]	Brain imaging	SVM	47	87.5
Ghiassian et al. 2016 [22]	Brain imaging	SVM	1111	65.0
Yahata et al. 2016 [23]	Brain imaging	Logistic regression	181	85.0
Chen et al. 2016 [24]	Brain imaging	SVM	240	79.2
Chanel et al. 2016 [25]	Brain imaging	SVM	29	92.3
Grossi et al. 2017 [26]	Brain imaging	Tree/forest	25	100.0
Guo et al. 2017 [27]	Brain imaging	Neural network	110	86.4
Kam et al. 2017 [28]	Brain imaging	Neural network	133	80.8
Subbaraju et al. 2017 [29]	Brain imaging	SVM	37	95.0
Ibrahim et al. 2018 [30]	Brain imaging	KNN	19	94.2
Zhang et al. 2018 [31]	Brain imaging	SVM	149	78.3
Bi at al. 2018 [32]	Brain imaging	SVM	84	96.2
Sen et al. 2018 [33]	Brain imaging	SVM	800	64.3
Dekhil et al. 2018 [34]	Brain imaging	SVM	283	91.0
Aghdam et al. 2018 [35]	Brain imaging	Neural network	185	65.6
Bhaumik et al. 2018 [36]	Brain imaging	Partial least square regression	372	62.0
Heinsfeld et al. 2018 [37]	Brain imaging	Neural network	1035	70.0
Kazeminejad et al. 2019 [38]	Brain imaging	SVM	51	95.0
Huang et al. 2019 [39]	Brain imaging	SVM	92	79.4
Payabvash et al. 2019 [40]	Brain imaging	Tree/forest	57	75.3
Kong et al. 2019 [41]	Brain imaging	Neural network	182	90.4
Feczko et al. 2018 [42]	Behavioural/ cognitive	Tree/forest	105	72.7
West et al. 2014 [43]	Clinical chemistry	SVM	82	81.0
Anwar et al. 2018 [44]	Clinical chemistry	SVM	69	89.0
Liu et al. 2016 [45]	Eye tracking	SVM	87	88.5
Wan et al. 2019 [46]	Eye tracking	Discriminant function analysis	74	85.1

Continued on next page

Supporting information: S1 File. – continued from previous page

Study	Modality	Classifier	Sample size	Accuracy, %
Latkowski & Osowski 2015 [47]	Gene expression	Tree/forest	146	78.4
Oh et al. 2017 [48]	Gene expression	SVM	42	93.8
Perego et al. 2009 [49]	Motion tracking	SVM	20	100.0
Crippa et al. 2015 [50]	Motion tracking	SVM	30	96.7
Anzulewicz et al. 2016 [51]	Motion tracking	Tree/forest	82	93.0
Li et al. 2018 [52]	Motion tracking	SVM	30	93.3
Ilias et al. 2018 [53]	Motion tracking	Neural network	44	98.4
Parikh, et al. 2019 [54]	Personal characteristic data	Neural network	851	62.0
Tariq et al. 2018 [55]	Video ratings	Logistic regression	162	80.3

References

1. Akshoomoff N, Lord C, Lincoln AJ, Courchesne RY, Carper RA, Townsend J, et al. Outcome classification of preschool children with autism spectrum disorders using MRI brain measures. Journal of the American Academy of Child & Adolescent Psychiatry. 2004 Mar;43(3):349-57.

2. Jiao Y, Chen R, Ke X, Chu K, Lu Z, Herskovits EH. Predictive models of autism spectrum disorder based on brain regional cortical thickness. Neuroimage. 2010 Apr;50(2):589-99.

3. Ecker C, Rocha-Rego V, Johnston P, Mourao-Miranda J, Marquand A, Daly EM, et al. Investigating the predictive value of whole-brain structural MR scans in autism: a pattern classification approach. Neuroimage. 2010 Jan;49(1):44-56.

4. Ecker C, Marquand A, Mourão-Miranda J, Johnston P, Daly EM, Brammer MJ, et al. Describing the brain in autism in five dimensions—magnetic resonance imaging-assisted diagnosis of autism spectrum disorder using a multiparameter classification approach. Journal of Neuroscience. 2010 Aug;30(32):10612-23.

5. Ingalhalikar M, Parker D, Bloy L, Roberts TP, Verma R. Diffusion based abnormality markers of pathology: toward learned diagnostic prediction of ASD. Neuroimage. 2011 Aug;57(3):918-27.

6. Anderson JS, Nielsen JA, Froehlich AL, DuBray MB, Druzgal TJ, Cariello AN, et al. Functional connectivity magnetic resonance imaging classification of autism. Brain. 2011 Oct;134(12):3742-54.

7. Uddin LQ, Menon V, Young CB, Ryali S, Chen T, Khouzam A, et al. Multivariate searchlight classification of structural magnetic resonance imaging in children and adolescents with autism. Biological Psychiatry. 2011 Nov;70(9):833-41.

8. Murdaugh DL, Shinkareva SV, Deshpande HR, Wang J, Pennick MR, Kana RK. Differential deactivation during mentalizing and classification of autism based on default mode network connectivity. PLoS ONE. 2012 Nov;7(11):e50064.

9. Uddin LQ, Supekar K, Lynch CJ, Khouzam A, Phillips J, Feinstein C, et al. Salience network–based classification and prediction of symptom severity in children with autism. JAMA Psychiatry. 2013 Aug;70(8):869-79.

10. Deshpande G, Libero L, Sreenivasan KR, Deshpande H, Kana RK. Identification of neural connectivity signatures of autism using machine learning. Frontiers in Human Neuroscience. 2013 Oct;7:670.

11. Just MA, Cherkassky VL, Buchweitz A, Keller TA, Mitchell TM. Identifying autism from neural representations of social interactions: neurocognitive markers of autism. PLoS ONE. 2014 Dec;9(12):e113879.

12. Wee CY, Wang L, Shi F, Yap PT, Shen D. Diagnosis of autism spectrum disorders using regional and interregional morphological features. Human Brain Mapping. 2014 Jul;35(7):3414-30.

13. Segovia F, Holt R, Spencer M, Górriz JM, Ramírez J, Puntonet CG, et al. Identifying endophenotypes of autism: a multivariate approach. Frontiers in Computational Neuroscience. 2014 Jun;8:60.

14. Zhou Y, Yu F, Duong T. Multiparametric MRI characterization and prediction in autism spectrum disorder using graph theory and machine learning. PLoS ONE. 2014 Jun;9(6):e90405.

15. Jamal W, Das S, Oprescu IA, Maharatna K, Apicella F, Sicca F. Classification of autism spectrum disorder using supervised learning of brain connectivity measures extracted from synchrostates. Journal of Neural Engineering. 2014 Jul;11(4):046019.

16. Chen CP, Keown CL, Jahedi A, Nair A, Pflieger ME, Bailey BA, et al. Diagnostic classification of intrinsic functional connectivity highlights somatosensory, default mode, and visual regions in autism. NeuroImage: Clinical. 2015 Jan;8:238-45.

17. Jin Y, Wee CY, Shi F, Thung KH, Ni D, Yap PT, et al. Identification of infants at high-risk for autism spectrum disorder using multiparameter multiscale white matter connectivity networks. Human Brain Mapping. 2015 Dec;36(12):4880-96

18. Plitt M, Barnes KA, Martin A. Functional connectivity classification of autism identifies highly predictive brain features but falls short of biomarker standards. NeuroImage: Clinical. 2015 Jan;7:359-66.

19. Iidaka T. Resting state functional magnetic resonance imaging and neural network classified autism and control. Cortex. 2015 Feb;63:55-67.

20. Libero LE, DeRamus TP, Lahti AC, Deshpande G, Kana RK. Multimodal neuroimaging based classification of autism spectrum disorder using anatomical, neurochemical, and white matter correlates. Cortex. 2015 May;66:46-59.

21. Li J, Qiu L, Xu L, Pedapati EV, Erickson CA, Sunar U. Characterization of autism spectrum disorder with spontaneous hemodynamic activity. Biomedical Optics Express. 2016 Oct;7(10):3871-81.

22. Ghiassian S, Greiner R, Jin P, Brown MR. Using functional or structural magnetic resonance images and personal characteristic data to identify ADHD and autism. PLoS ONE. 2016 Dec;11(12):e0166934.

23. Yahata N, Morimoto J, Hashimoto R, Lisi G, Shibata K, Kawakubo Y, et al. A small number of abnormal brain connections predicts adult autism spectrum disorder. Nature Communications. 2016 Apr;7:11254.

24. Chen H, Duan X, Liu F, Lu F, Ma X, Zhang Y, et al. Multivariate classification of autism spectrum disorder using frequency-specific resting-state functional connectivity—a multi-center study. Progress in Neuro-Psychopharmacology and Biological Psychiatry. 2016 Jan;64:1-9.

25. Chanel G, Pichon S, Conty L, Berthoz S, Chevallier C, Grèzes J. Classification of autistic individuals and controls using cross-task characterization of fMRI activity. NeuroImage: Clinical. 2016 Jan;10:78-88.

26. Grossi E, Olivieri C, Buscema M. Diagnosis of autism through EEG processed by advanced computational algorithms: a pilot study. Computer Methods and Programs in Biomedicine. 2017 Apr;142:73-9.

27. Guo X, Dominick KC, Minai AA, Li H, Erickson CA, Lu LJ. Diagnosing autism spectrum disorder from brain resting-state functional connectivity patterns using a deep neural network with a novel feature selection method. Frontiers in Neuroscience. 2017 Aug;11:460.

28. Kam TE, Suk HI, Lee SW. Multiple functional networks modeling for autism spectrum disorder diagnosis. Human Brain Mapping. 2017 Nov;38(11):5804-21.

29. Subbaraju V, Suresh MB, Sundaram S, Narasimhan S. Identifying differences in brain activities and an accurate detection of autism spectrum disorder using resting state functional-magnetic resonance imaging: A spatial filtering approach. Medical Image Analysis. 2017 Jan;35:375-89.

30. Ibrahim S, Djemal R, Alsuwailem A. Electroencephalography (EEG) signal processing for epilepsy and autism spectrum disorder diagnosis. Biocybernetics and Biomedical Engineering. 2018 Jan;38(1):16-26.

31. Zhang F, Savadjiev P, Cai W, Song Y, Rathi Y, Tunç B, et al. Whole brain white matter connectivity analysis using machine learning: an application to autism. Neuroimage. 2018 May;172:826-37.

32. Bi XA, Wang Y, Shu Q, Sun Q, Xu Q. Classification of autism spectrum disorder using random support vector machine cluster. Frontiers in Genetics. 2018 Feb;9:18.

33. Sen B, Borle NC, Greiner R, Brown MR. A general prediction model for the detection of ADHD and Autism using structural and functional MRI. PLoS ONE. 2018 Apr;13(4):e0194856.

34. Dekhil O, Hajjdiab H, Shalaby A, Ali MT, Ayinde B, Switala A, et al. Using resting state functional MRI to build a personalized autism diagnosis system. PLoS ONE. 2018 Oct;13(10):e0206351.

35. Aghdam MA, Sharifi A, Pedram MM. Combination of rs-fMRI and sMRI data to discriminate autism spectrum disorders in young children using deep belief network. Journal of Digital Imaging. 2018 Dec;31(6):895-903.

36. Bhaumik R, Pradhan A, Das S, Bhaumik DK. Predicting autism spectrum disorder using domain-adaptive cross-site evaluation. Neuroinformatics. 2018 Apr;16(2):197-205.

37. Heinsfeld AS, Franco AR, Craddock RC, Buchweitz A, Meneguzzi F. Identification of autism spectrum disorder using deep learning and the ABIDE dataset. NeuroImage: Clinical. 2018 Jan;17:16-23.

38. Kazeminejad A, Sotero RC. Topological properties of resting-state fMRI functional networks improves machine learning-based autism classification. Frontiers in Neuroscience. 2018;12:1018.

39. Huang H, Liu X, Jin Y, Lee SW, Wee CY, Shen D. Enhancing the representation of functional connectivity networks by fusing multi-view information for autism spectrum disorder diagnosis. Human Brain Mapping. 2019 Feb;40(3):833-54.

40. Payabvash S, Palacios EM, Owen JP, Wang MB, Tavassoli T, Gerdes M, et al. White matter connectome edge density in children with autism spectrum disorders: potential imaging biomarkers using machine-learning models. Brain Connectivity. 2019 Mar;9(2):209-20.

41. Kong Y, Gao J, Xu Y, Pan Y, Wang J, Liu J. Classification of autism spectrum disorder by combining brain connectivity and deep neural network classifier. Neurocomputing. 2019 Jan;324:63-8.

42. Feczko E, Balba NM, Miranda-Dominguez O, Cordova M, Karalunas SL, Irwin L, et al. Subtyping cognitive profiles in autism spectrum disorder using a functional random forest algorithm. Neuroimage. 2018 May;172:674-88.

43. West PR, Amaral DG, Bais P, Smith AM, Egnash LA, Ross ME, et al. Metabolomics as a tool for discovery of biomarkers of autism spectrum disorder in the blood plasma of children. PLoS ONE. 2014 Nov;9(11):e112445.

44. Anwar A, Abruzzo PM, Pasha S, Rajpoot K, Bolotta A, Ghezzo A, et al. Advanced glycation endproducts, dityrosine and arginine transporter dysfunction in autism-a source of biomarkers for clinical diagnosis. Molecular Autism. 2018 Dec;9(1):3.

45. Liu W, Li M, Yi L. Identifying children with autism spectrum disorder based on their face processing abnormality: A machine learning framework. Autism Research. 2016 Aug;9(8):888-98.

46. Wan G, Kong X, Sun B, Yu S, Tu Y, Park J, et al. Applying Eye Tracking to Identify Autism Spectrum Disorder in Children. Journal of Autism and Developmental Disorders. 2019 Jan;49(1):209-15.

47. Latkowski T, Osowski S. Data mining for feature selection in gene expression autism data. Expert Systems with Applications. 2015 Feb 1;42(2):864-72.

48. Oh DH, Kim IB, Kim SH, Ahn DH. Predicting autism spectrum disorder using blood-based gene expression signatures and machine learning. Clinical Psychopharmacology and Neuroscience. 2017 Feb;15(1):47.

49. Perego P, Forti S, Crippa A, Valli A, Reni G. Reach and throw movement analysis with support vector machines in early diagnosis of autism. In2009 Annual International Conference of the IEEE Engineering in Medicine and Biology Society 2009 Sep (pp. 2555-2558). IEEE.

50. Crippa A, Salvatore C, Perego P, Forti S, Nobile M, Molteni M, et al. Use of machine learning to identify children with autism and their motor abnormalities. Journal of Autism and Developmental Disorders. 2015 Jul;45(7):2146-56.

51. Anzulewicz A, Sobota K, Delafield-Butt JT. Toward the autism motor signature: Gesture patterns during smart tablet gameplay identify children with autism. Scientific Reports. 2016 Aug;6:31107.

52. Li B, Sharma A, Meng J, Purushwalkam S, Gowen E. Applying machine learning to identify autistic adults using imitation: An exploratory study. PLoS ONE. 2017 Aug;12(8):e0182652.

53. Ilias S, Tahir NM, Jailani R, Hasan CZ. Linear Discriminant Analysis in Classifying Walking Gait of Autistic Children. In2017 European Modelling Symposium (EMS) 2017 Nov (pp. 67-72). IEEE.

54. Parikh MN, Li H, He L. Enhancing diagnosis of autism with optimized machine learning models and personal characteristic data. Frontiers in Computational Neuroscience. 2019;13:9.

55. Tariq Q, Daniels J, Schwartz JN, Washington P, Kalantarian H, Wall DP. Mobile detection of autism through machine learning on home video: A development and prospective validation study. PLoS Medicine. 2018 Nov;15(11):e1002705.
